# Vitamin D deficiency in Ukraine: A multicentre cross-sectional study

**DOI:** 10.1016/j.gloepi.2024.100170

**Published:** 2024-10-10

**Authors:** Sofiia Shatylo, Volodymyr Bogomaz, Oleksii Babych

**Affiliations:** a Department of Internal Medicine, Faculty of Dentistry, Bogomolets National Medical University, 9 Volodymyr Vynnychenko Str., 04053 Kyiv, Ukraine; b Department оf Modern Technologies оf Medical Diagnostics and Treatment, Bogomolets National Medical University, 34 Beresteiskyi Ave., 03057 Kyiv, Ukraine

**Keywords:** Vitamin D, 25-hydroxyvitamin D, Vitamin D deficiency, Vitamin D insufficiency, Ukraine

## Abstract

**Background:**

Available epidemiological data on vitamin D status in the Ukrainian population are limited.

**Objective:**

The aim of this study is to investigate the prevalence of vitamin D deficiency in Ukraine.

**Methods:**

This multicentre cross-sectional study included a total of 11,462 participants: 1530 children with a median age 10 years, (IQR 6–14) and 56.21 % of them were female; 9932 adults with a median age of 36 years (IQR 26–48) and 78.72 % of them were female. Serum 25-hydroxyvitamin D (25(OH)D) levels were measured once by chemiluminescent immunoassay (UniCel DxI 800 Access Immunoassay System, Beckman Coulter Inc., USA) in the period of January–December 2021 in Kyiv and Kyiv Oblast. The cut-offs were: vitamin D deficiency, <50 nmol/L; moderate deficiency, 25–<50 nmol/L; severe deficiency, <25 nmol/L; vitamin D insufficiency, 50–<75 nmol/L; vitamin D sufficiency, 75–<250 nmol/L; optimal concentration, 75–<125 nmol/L; increased levels, 125–<250 nmol/L; and toxicity, ≥250 nmol/L.

**Results:**

The median 25(OH)D level was 65.4 nmol/L (IQR 49.5–85.6) among all participants, severe vitamin D deficiency was recorded in 2.9 % of participants (95 % CI: 2.6–3.2), moderate deficiency in 23 % (95 % CI: 22–24), and vitamin D insufficiency in 37 % (95 % CI: 36–38).

Prevalence of vitamin D deficiency in group 1–17 years was 23.5 % (95 % CI: 21–26). We did not find vitamin D deficiency in children aged 1–2 years; however toxic levels were recorded in 4.2 % of the children in this age group (95 % CI: 1.4–9.6). Among the adults (≥18 years old), prevalence of vitamin D deficiency was 26 % (95 % CI: 25–27).

**Conclusion:**

Vitamin D deficiency and insufficiency are common in Ukraine.

## Introduction

Vitamin D deficiency poses a significant public health challenge in many countries. It is not only associated with deficiency diseases, such as rickets, but is also linked to a variety of prevalent chronic conditions in adulthood, including diabetes, cancer, infectious diseases, cardiovascular issues, and autoimmune disorders [1]. Due to vitamin D's function in regulating the immune system, its role also was studied during the COVID-19 pandemic [2]. Researches in many countries [[3], [4], [5]], including a study conducted in Ukraine [6], has analysed the possible impact of serum 25-hydroxyvitamin D (25(OH)D) levels on COVID-19 outcomes and the results of a recent meta-analysis suggest that vitamin D3 supplementation may potentially reduce the risk of negative outcomes, such as intensive care unit admissions and death [7].

The availability of data on the prevalence of vitamin D deficiency may help to identify possible associations and causalities between vitamin D and other diseases and their outcomes.

Serum 25(OH)D levels, which are considered to reflect vitamin D status, may be adequate in some countries; however, vitamin D deficiency remains prevalent in many countries [8].

As it was mentioned in review by Lips P. et al., vitamin D deficiency prevails in both Europe and the Middle East with severe deficiency (serum 25(OH)D < 30 nmol/L or 12 ng/mL) found in >10 % of Europeans [9]. During the last decade, some studies were conducted in Ukraine, mostly including adults and most of them performed by a researchers' group from one medical centre providing highly specialized medical care [[10], [11], [12]]. There is still a need for large studies involving both children and adults, performed by independent researchers, to provide additional information about the prevalence of vitamin D deficiency in Ukraine, making this study relevant and vital.

## Materials and methods

This was a multicentre cross-sectional study. The inclusion criteria: children and adults aged 1 year and older, in whom serum vitamin D concentrations were collected once during January–December 2021 in Kyiv and Kyiv Oblast in 96 different medical offices, and who provided informed consent at the time of primary data collection. 25(OH)D levels were measured by chemiluminescent immunoassay in central regional laboratory office. Serum 25(OH)D levels were measured by chemiluminescent immunoassay in central regional laboratory office (UniCel DxI 800 Access Immunoassay System, Beckman Coulter Inc., USA). This assay exhibits total imprecision ≤10.0 % at concentrations greater than 37.5 nmol/L, and total Standard Deviation (SD) ≤ 3.8 nmol/L at concentrations ≤37.5 nmol/L. The manufacturer's data has been confirmed by the laboratory in the verification procedure. The precision was: CV within-run = 7 %, CV between-run = 6 %. Laboratory long-term precision is ≤9.6 %. The accuracy was evaluated by the manufacturer in comparison with liquid chromatography coupled with tandem mass spectrometry (LC-MS/MS) method and gave the following results (Passing-Bablok regression and Pearson correlation): *r* = 0.91, y = 0.95*x-2.23 (nmol/L). Bias is −11 % at concentrations = 37.5 nmol/L. Estimating bias then was verified relative to the peer group (*n* = 108) for External Quality Assessment (EQA) programs and was 5 %. Laboratory is certificated by ISO 9001:2015 and participates in comprehensive international quality assurance programs. Demographic data such as age and sex were recorded. Grouping participant's by age is still remaining challenging. As it was mention by Diaz et al. [13] there is a great variability in age groupings used to record and report health data due to the lack of standard age categories. The National Institutes of Health (NIH) recommended to use a specific age or age range, if possible [14]. Hence, we use Age Stages Defined According to NICHD Paediatric Terminology [15] for participants from 1 year to 17 years old and classified them into 4 groups: toddler (1–2 years), early childhood (3–5 years), middle childhood (6–11 years), early adolescence (12–17 years). Based on the National Institutes of Health (NIH) recommendations and recommended the American Medical Associations' age designations [14] participants 18 years and older were considered as adults and classified into 5 groups based on the Medical Subject Headings (MeSH) which is the National Library of Medicine's controlled vocabulary thesaurus (Medical Subject Headings (Mesh): young adult (18–24 years), adult (25–44 years), middle-age (45–64 years), elderly (65–79 years), 80 and over (≥80 years) [16].

As it was mentioned in review by Roger Bouillon, there are a lot of different approaches to define vitamin D deficiency and insufficiency [17]. Therefore, we formed main groups based on an Endocrine Society Clinical Practice Guideline [18] and subgroups based on Guidelines for Preventing and Treating Vitamin D Deficiency: A 2023 Update in Poland [19].

Hence, the cut-offs for 25(OH)D levels were defined as follows:

• Vitamin D deficiency as <50 nmol/L (<20 ng/mL) with further subdivision to moderate deficiency as 25- < 50 nmol/L (10- < 20 ng/mL) and severe deficiency as <25 nmol/L (<10 ng/mL);
• Vitamin D insufficiency (suboptimal status) as 50–<75 nmol/L (20–<30 ng/mL);
• Vitamin D sufficiency as 75- < 250 nmol/L (30- < 100 ng/mL) with further subdivision to optimal concentration 75–<125 nmol/L (30–<50 ng/mL) and increased levels 125- < 250 nmol/L (50–100 ng/mL)
• Toxicity as ≥250 nmol/L (≥100 ng/mL).

### Statistical analysis

The necessary sample size (*n* = 350) was defined using the formula for a prevalence survey [20] with precision 5 %, expected prevalence 35 % as was found in previous studies in Ukraine [10], and 95 % Level of the confidence interval.

The Kolmogorov–Smirnov test was used to check if the distribution of continuous data was normal. Therefore, we expressed continuous variables as median values with the interquartile range (IQR), as most data did not follow a normal distribution. To assess the correlation between continuous variables, we used both the Pearson correlation coefficient and Spearman's rank correlation coefficient (rho). For the Pearson correlation, we performed log transformation to approximate a normal distribution. Categorical variables are presented as the number of cases and percentages. We used the chi-square test to compare the categorical data. Confidence intervals for proportions were calculated using the binomial “exact” calculation. We presented the prevalence with 95 % confidence intervals (CI). Statistical significance was defined as *p* < 0.05. Data were analysed using MedCalc® Statistical Software version 20.215 [21].

## Results

We screened 11,549 participants for eligibility and eighty-seven of them were excluded as they were aged less than one year. As a result, 11,462 participants were included in data analysis with age median 36 years (IQR 26–48) and 75.71 % of them were female (*n* = 8678). Age median in children was 10 years (IQR 6–14) and in adults – 38 years (IQR 30–50). Detailed age and sex distribution are shown in Table 1.

**Table 1:** Age and sex distribution of the participants.

	Both sexes, n	Males, n	Females, n	Males, %	Females, %
All participants	11,462	2784	8678	24.29	75.71
Children and teenagers (1–17 years)	1530	670	860	43.79	56.21
1–2 years	119	73	46	61.34	38.66
3–5 years	228	119	109	52.19	47.81
6–11 years	546	252	294	46.15	53.85
12–17 years	637	226	411	35.48	64.52
Adults (≥18 years)	9932	2114	7818	21.28	78.72
18–24 years	943	157	786	16.65	83.35
25–44 years	5461	975	4486	17.85	82.15
45–64 years	2757	765	1992	27.75	72.25
65–79 years	701	188	513	26.82	73.18
≥ 80 years	70	29	41	41.43	58.57

Median of vitamin D level was 65.4 nmol/L (IQR 49.5–85.6) among all participants. Median of 25(OH)D level in children and teenagers (aged 1–17 years) was 68 nmol/L (IQR 51.2–90.7); 72.35 nmol/L (IQR 55.4–96.3) for males and 65.15 nmol/L (IQR 48.25–84.4) for females respectively. Median of vitamin D level in adults (≥18 years) – 65 nmol/L (IQR 49.2–85). Median of 25(OH)D level in women (≥18 years) was 65.3 nmol/L (IQR 49.7–84.8) and median of 25(OH)D level in men (≥18 years) - 65.3 nmol/L (IQR 48.5–88.55) respectively. Detailed information about 25(OH)D levels in different age and sex groups are summarized in Table 2.

**Table 2:** Serum vitamin D levels (25(OH)D) in different age and sex groups.

Age	Serum 25(OH)D levels⁎		
	All	Males	Females
1-100 years	65.4 (49.5–85.6)	65.6 (48.5–88.6)	65.3 (49.7–84.8)
Children and teenagers (1–17 years)	68 (51.2–90.7)	72.35 (55.4–96.3)	65.15 (48.25–84.4)
1–2 years	122 (87.13–160)	122 (88.4–154.25)	127 (84.4–169)
3–5 years	83.45 (62.4–105.5)	81.9 (61.5–104)	85 (63.05–108)
6–11 years	68.9 (54.5–89.4)	73.7 (57.35–92.65)	65.75 (51.2–83.6)
12–17 years	58.9 (43.08–75.53)	61 (45.4–76)	57 (41.7–75)
Adults (≥18 years)	65 (49.2–85)	63.6 (47.1–85.9)	65 (49.9–84.8)
18–24 years	56.9 (42.225–75.375)	54.5 (40.6–71.5)	57.85 (42.9–76.1)
25–44 years	65.8 (50.5–85.5)	61.9 (46.1–83)	66.5 (51.4–85.8)
45–64 years	66.2 (50.175–86.6)	65.4 (48.575–87.325)	66.3 (50.55–86.25)
65–79 years	63.7 (46.175–85.45)	68.65 (51.1–94.6)	62.9 (45.6–82.475)
≥ 80 years	63.45 (46.7–89.4)	67.9 (56.425–112)	61.2 (40.075–71.35)

⁎ Note. Data are median, (IQR), nmol/L.

We found a very weak negative correlation between age and serum 25(OH)D level - the Pearson correlation coefficient *r* = −0.076 (95 % CI, −0.09402 to −0.05761, *p* < 0.001), log-transformed data. We additionally calculated Spearman's rank correlation coefficient (rho) for the same variables; however, we did not find evidence to support the association between age and serum 25(OH)D level (rho = 0.00971, 95 % CI: −0.00860 to 0.0280, *p* = 0.2988).

After grouping participants according to their serum 25(OH)D levels, we found that the prevalence of severe vitamin D deficiency among all participants was 2.9 % (95 % CI: 2.6–3.2). Among all participants, in males prevalence of severe vitamin D deficiency was 4.5 % (95 % CI: 3.7–5.3) and in females - 2.4 %, (95 % CI: 2.1–2.7). Among all participants moderate deficiency was recorded in 23 % (95 % CI, 22–24) and vitamin D insufficiency in 37 % (95 % CI, 36–38).

**Fig. 1:**
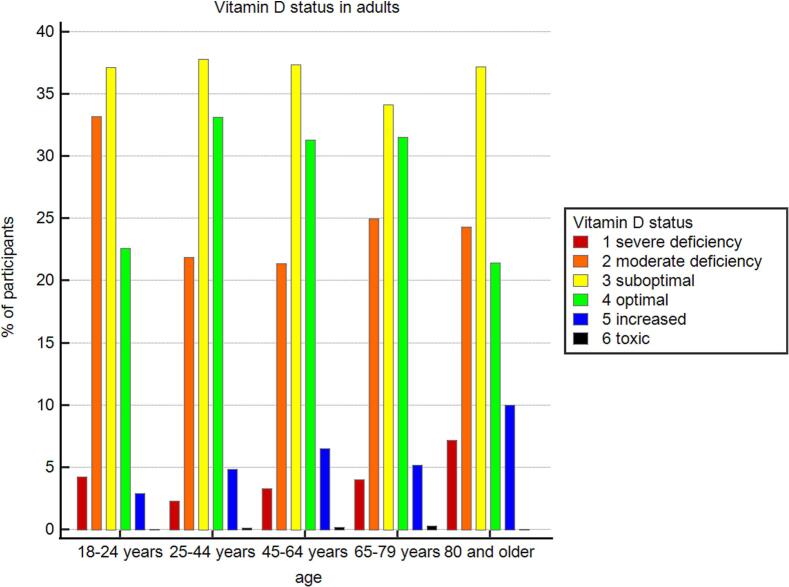
Vitamin D status in adults.

The distribution of vitamin D status in adults was following: severe deficiency - 2.9 % (95 % CI: 2.6–3.3), moderate deficiency - 23 % (95 % CI: 22–24), suboptimal levels - 37 % (95 % CI: 36–38), optimal levels - 31 % (95 % CI: 30.5–32.3), increased levels - 5.2 % (95 % CI: 4.7–5.6), toxic levels - 0.1 % (95 % CI: 0.08–0.24). In adults, the prevalence of severe vitamin D deficiency was 5.3 % (95 % CI: 4.4–6.3) in males and 2.3 % (95 % CI: 1.9–2.6) in females. More detailed data are summarized in Fig. 1 and Supplementary materials.

**Fig. 2:**
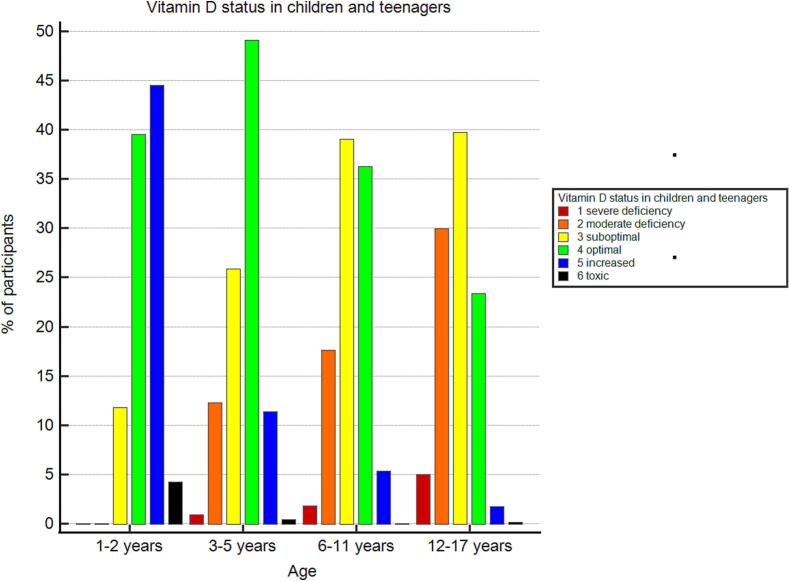
Vitamin D status in children and teenagers.

The distribution of vitamin D status in children and teenagers (1–17 years) was the following: severe deficiency - 2.9 % (95 % CI: 2.1–3.8), moderate deficiency - 21 % (95 % CI: 19–23), suboptimal levels - 35 % (95 % CI: 33–38), optimal levels - 33 % (95 % CI: 31–36), increased levels - 7.8 % (95 % CI: 6.5–9.2), toxic levels - 0.5 % (95 % CI: 0.2–0.9). Among children and teenagers, the prevalence of severe vitamin D deficiency was 1.8 % (95 % CI: 0.9–3.1) in males and 3.7 % (95 % CI: 2.6–5.2) in females. We found that children aged 1–2 years did not have vitamin D deficiency; however, suboptimal levels were recorded in 12 % of children in that age group (95 % CI: 7–19). More detailed data are presented in Fig. 2 and Supplementary materials.

## Discussion

Our study found that vitamin D deficiency and insufficiency are widespread in Ukraine and notably affect teenagers and adults. Using the PubMed databases, we found some studies conducted in the Ukrainian population. Our outcomes are slightly different from those published by Grygorieva NV in 2023, a single-centre study conducted between 2016 and 2022 years in the same region of Ukraine [12]. To be more precise we compare median of serum vitamin D obtained during 2021 year by both studies: median of vitamin D level in our study was 65 nmol/L ([IQR 49.2–85], ≥18 years, 9932 participants), which is lower than 35.0 ng/mL ([IQR 26.8–45.8] = 87.36 nmol/L, [IQR 66.9–114.3], ≥20 years, 805 participants). This may be due to differences in age and sex distribution. Comparing by sex shown the similar difference: median of 25(OH)D level in women (≥18 years, 7818 participants) in our work was 65.3 nmol/L (IQR 49.7–84.8) vs 35.5 ng/mL ([IQR 27.0–46.4] = 88.6 nmol/L [IQR 67.4–115.8], ≥20 years, 704 participants). And in men (≥18 years, 2114 participants) in our study median was 65.3 nmol/L (IQR 48.5–88.55), which is also lower than in Grygorieva NV's work, where men's median was 31.4 ng/mL, respectively ([IQR 24.2–39.8] = 78.4 nmol/L [IQR 60.4–99.3], ≥20 years, 101 participants). However, collation by age groups was not possible to perform as in Grygorieva NV's study different age categorizing was used. Analysis of vitamin D deficiency (serum levels <50 nmol/L / <20 ng/mL) prevalence among adults ≥18 years in our study showed 26 % (95 % CI: 25–27), which is higher than 9.7 % reported by Grygorieva NV in the same year (2021). However, the data of previous years (2016–2017) in that study showed other findings at 33.7 % and 25.3 %, respectively.

In another work published by Shchubelka K., who studied the prevalence of vitamin D deficiency in 2019 year in Transcarpathia region of Ukraine, the mean ± SD of serum vitamin D in adults (1639 participants) was 22.67 ± 8.63 ng/mL, which is approximately 56.58 nmol/L and similar to our results [10]. As the prevalence of vitamin D deficiency was reported for different age groups, and it was possible to compare data for the group aged 18–24 years: 36.2 % (*n* = 232) reported by Shchubelka K., which corresponds to our outcomes: 37 % (95 % CI: 34–40, *n* = 943). Although the blood sample was collected only from 23 infants and toddlers aged 0–3 years, the author did not observe vitamin D deficiency (25(OH)D < 50 nmol/L/<20 ng/mL) in that age group, similar to the results of our study. The prevalence of vitamin D deficiency in teenagers (13–17 years, *n* = 81) was 35.8 %, which is consistent with our results, showing a prevalence of 35 % (95 % CI: 0.31–39; *n* = 637).

In the recent research by Shanyhin A. et al. 928 residents of the southern region of Ukraine aged from 19 to 82 were examined [11]. The authors established that 33.6 % of the participants had vitamin D deficiency (serum 25(OH)D levels <50 nmol/L ≤20 ng/mL). Vitamin D insufficiency (defined as 51–74 nmol/l = 21–29 ng/ml) and sufficiency (≥75 nmol/L = ≥30 ng/mL) were observed in 33 % and 33.4 % respectively.

As there is some hypothesis regarding latitude influence to prevalence of vitamin D deficiency [22,23], we compared our results to outcomes of the studies conducted in European countries located on the same latitude as Ukraine. For example, a study in France [24] showed similar results with 34.6 % a prevalence of vitamin D deficiency (25(OH)D < 50 nmol/L/<20 ng/mL) in adults (*n* = 892, aged 18–89 years); however, the prevalence of severe deficiency defined as <25 nmol/L(<10 ng/mL) was 6.3 %, which is higher than our findings of 2.9 % (95 % CI: 2.6–3.3, *n* = 9932, aged 18–100 years). In a study conducted in Germany in 2015, a higher prevalence of vitamin D deficiency was reported; 61.6 % of the participants had serum 25(OH)D levels <50 nmol/l and 30.2 % had levels <30 nmol/l. [25] Large Polish study published in 2016 year shown also higher prevalence of serum levels of 25(OH)D less than 20 ng/mL – near 65.8 % (*n* = 5775) [26].

## Limitations

Unfortunately, it was not possible to obtain information about the participants' use of vitamin D supplements. The cost of measuring 25(OH)D was not covered by the national healthcare program; therefore, we may predict lower 25(OH)D levels in the population, as some categories might not attend private medical laboratories to carry out blood tests. Considering the available data on positive migration rates to Kyiv city and Kyiv oblast from other regions of the country in 2021 (according to the Main Statistics Office of Kyiv city and Kyiv oblast [27,28]), the blood sample collection from various medical offices, and our large sample size, we suggest that the obtained results reflect the average results for the country. However, we acknowledge a possible limitation in generalisability, as we did not collect data from other regions of the country.

## Conclusions

To the best of our knowledge, this is the largest study conducted in Ukraine and provides comprehensive information about vitamin D status in this population.

Vitamin D deficiency and insufficiency are common in Ukraine, especially in teenagers and adults. Approximately 63 % (95 % CI: 62–64) of the population did not achieve optimal levels of serum vitamin D levels, among which severe deficiency was recorded in 2.9 % (95 % CI: 2.6–3.2), moderate deficiency in 23 % (95 % CI: 22–24), and vitamin D insufficiency in 37 % (95 % CI: 36–38).

Medical practitioners should warn parents of the importance of using age-appropriate dosages of vitamin D supplements in small children to avoid the risk of overdose. In addition, laboratory testing for serum vitamin D (25(OH)D) seems to be reasonable in this age group to detect possible vitamin D overdose, if suspected. Teenagers and adults should be encouraged to spent more time outside to make possible body synthesis of vitamin D. Medical practitioners can advise usage of prophylactic doses of vitamin D as recommended by current guidelines and may offer laboratory testing for serum vitamin D levels (25(OH)D) as due to high prevalence of vitamin D deficiency some patient may need higher dosages of vitamin D.

## Funding

This study did not receive any specific grant from public, commercial or not-for-profit funding agencies.

## Ethical statement

The study was conducted according to the guidelines laid down in the Declaration of Helsinki and was approved by the Ethic Committee of the Bogomolets National Medical University, Kyiv, Ukraine (Protocol number 183). All patients and/or their representatives gave informed consent during the primary data collection. For statistical analysis, properly anonymised datasets were used.

## AI statement

The authors confirm no generative AI or AI-assisted technology was used to generate content.

## CRediT authorship contribution statement

**Sofiia Shatylo:** Writing – original draft, Methodology, Formal analysis, Conceptualization. **Volodymyr Bogomaz:** Writing – review & editing, Methodology, Data curation, Conceptualization. **Oleksii Babych:** Resources, Investigation, Data curation.

## Declaration of competing interest

The authors declare that they have no known competing financial interests or personal relationships that could have appeared to influence the work reported in this paper.

## Appendix A Supplementary data

Supplementary material. Table 3. Vitamin D status in different age and sex groups Image 1
