# Supplementary material for: Vitamin D deficiency in Ukraine: A multicentre cross-sectional study
Source: Glob Epidemiol. 2024 Oct 10;8:100170. doi: 10.1016/j.gloepi.2024.100170 (PMC11525160; doi:10.1016/j.gloepi.2024.100170)
Supplement: Supplementary file 1 — Supplementary material. Table 3. Vitamin D status in different age and sex groups [file mmc1.pdf]

Supplementary materials. Table 3. Vitamin D status in different age and sex groups

Group	Vitamin D deficiency			Vitamin D insufficiency (suboptimal status)	Vitamin D sufficiency			Toxicity	Total, n	p
	Severe deficiency <25 nmol/L (<10 ng/mL) n, PR*, 95% CI	Moderate deficiency 25-50 nmol/L (10-20 ng/mL) n, PR*, 95% CI	Deficiency <50 nmol/L (<20 ng/mL) (total) n, PR*, 95% CI	50–75 nmol/L (20-30 ng/mL) n, PR*, 95% CI	Optimal 75–125 nmol/L (30-50 ng/mL) n, PR*, 95% CI	Increased levels 125-250 nmol/L (50-100 ng/mL) n, PR*, 95% CI	75-250 nmol/L 30-100 ng/mL (total) n, PR*, 95% CI	>250 nmol/L (>100 ng/mL) n, PR*, 95% CI		
All participants	332<br>0.029<br>0.026-0.032	2603<br>0.23<br>0.22-0.24	2935<br>0.256<br>0.248-0.264	4247<br>0.37<br>0.36-0.38	3627<br>0.32<br>0.31-0.33	632<br>0.055<br>0.051-0.06	4259<br>0.37<br>0.36-0.38	21<br>0.002<br>0.001-0.003	11462	
Males	124<br>0.045<br>0.037-0.053	622<br>0.22<br>0.21-0.24	746<br>0.27<br>0.25-0.29	962<br>0.35<br>0.33-0.36	876<br>0.32<br>0.3-0.33	193<br>0.07<br>0.06-0.08	1069<br>0.38<br>0.37-0.4	7<br>0.003<br>0.001-0.005	2784	**<0.001**
Females	208<br>0.024<br>0.021-0.027	1981<br>0.23<br>0.22-0.24	2189<br>0.25<br>0.24-0.26	3285<br>0.38<br>0.37-0.39	2751<br>0.32<br>0.31<br>to 0.33	439<br>0.051<br>0.046-0.055	3190<br>0.37<br>0.36-0.38	14<br>0.002<br>0.001-0.0027	8678	
Children and teenagers, 1-17 years	44<br>0.029<br>0.021-0.038	315<br>0.21<br>0.19-0.23	359<br>0.235<br>0.21-0.26	539<br>0.35<br>0.33-0.38	506<br>0.33<br>0.31-0.36	119<br>0.078<br>0.065-0.092	625<br>0.41<br>0.38-0.43	7<br>0.005<br>0.002-0.009	1530	
Males, 1-17 years	12<br>0.018<br>0.009-0.031	117<br>0.18<br>0.15-0.21	129<br>0.19<br>0.16-0.23	224<br>0.33<br>0.3-0.37	247<br>0.37<br>0.33-0.41	65<br>0.097<br>0.076-0.122	312<br>0.47<br>0.43-0.5	5<br>0.008<br>0.002-0.017	670	**<0.001**
Females, 1-17 years	32<br>0.037<br>0.026-0.052	198<br>0.23<br>0.203-0.26	230<br>0.27<br>0.24-0.3	315<br>0.37<br>0.33-0.4	259<br>0.3<br>0.27-0.33	54<br>0.063<br>0.048-0.081	313<br>0.36<br>0.33-0.4	2<br>0.002<br>0.0003-0.008	860	
1-2 years	0	0	0	14<br>0.12<br>0.07-0.19	47<br>0.4<br>0.3-0.49	53<br>0.45<br>0.35-0.54	100<br>0.84<br>0.684-1.022	5<br>0.042<br>0.014-0.096	119	
Males, 1-2 years	0	0	0	7<br>0.096<br>0.039-0.19	32<br>0.44<br>0.32-0.56	30<br>0.41<br>0.3-0.53	62<br>0.85<br>0.75-0.92	4<br>0.06<br>0.015-0.134	73	0.4
Females, 1-2 years	0	0	0	7<br>0.152<br>0.063-0.28.9	15<br>0.33<br>0.2-0.48	23<br>0.5<br>0.35-0.65	38<br>0.83<br>0.69-0.92	1<br>0.022<br>0.0005-0.115	46	
3-5 years	2<br>0.009<br>0.001-0.031	28<br>0.12<br>0.08-0.17	30<br>0.13<br>0.09-0.18	59<br>0.26<br>0.2-0.32	112<br>0.49<br>0.42-0.56	26<br>0.11<br>0.08-0.16	138<br>0.61<br>0.54-0.67	1<br>0.004<br>0.0001-0.024	228	
Males, 3-5 years	1<br>0.008<br>0.0002-0.046	19<br>0.16<br>0.098-0.24	20<br>0.17<br>0.11-0.25	29<br>0.24<br>0.17-0.33	58<br>0.49<br>0.39-0.58	12<br>0.1<br>0.05-0.17	70<br>0.59<br>0.49-0.68	0	119	0.5

Group	Vitamin D deficiency			Vitamin D insufficiency (suboptimal status)	Vitamin D sufficiency			Toxicity	Total, n	p
	Severe deficiency <25 nmol/L (<10 ng/mL) n, PR*, 95% CI	Moderate deficiency 25-50 nmol/L (10-20 ng/mL) n, PR*, 95% CI	Deficiency <50 nmol/L (<20 ng/mL) (total) n, PR*, 95% CI	50–75 nmol/L (20-30 ng/mL) n, PR*, 95% CI	Optimal 75–125 nmol/L (30-50 ng/mL) n, PR*, 95% CI	Increased levels 125-250 nmol/L (50-100 ng/mL) n, PR*, 95% CI	75-250 nmol/L 30-100 ng/mL (total) n, PR*, 95% CI	>250 nmol/L (>100 ng/mL) n, PR*, 95% CI		
Females, 3-5 years	1<br>0.0092<br>0.0002-0.05	9<br>0.083<br>0.038-0.15	10<br>0.092<br>0.045-0.16	30<br>0.28<br>0.19-0.37	54<br>0.495<br>0.4-0.6	14<br>0.13<br>0.07-0.21	68<br>0.62<br>0.53-0.71	1<br>0.009<br>0.0002-0.05	109	
6-11 years	10<br>0.018<br>0.009-0.033	96<br>0.18<br>0.14-0.21	106<br>0.19<br>0.16-0.23	213<br>0.39<br>0.35-0.43	198<br>0.3626<br>0.32-0.4	29<br>0.053<br>0.036-0.075	227<br>0.42<br>0.37-0.46	0	546	
Males, 6-11 years	3<br>0.012<br>0.003-0.034	34<br>0.13<br>0.095-0.183	37<br>0.15<br>0.11-0.2	92<br>0.37<br>0.31-0.43	105<br>0.42<br>0.36-0.48	18<br>0.071<br>0.043-0.11	123<br>0.49<br>0.43-0.55	0	252	**0.01**
Females, 6-11 years	7<br>0.024<br>0.01-0.05	62<br>0.21<br>0.17-0.26	69<br>0.23<br>0.19-0.29	121<br>0.41<br>0.35-0.47	93<br>0.32<br>0.26-0.37	11<br>0.038<br>0.019-0.066	104<br>0.35<br>0.29-0.41	0	294	
12-17 years	32<br>0.05<br>0.035-0.07	191<br>0.3<br>0.26-0.34	223<br>0.35<br>0.31-0.39	253<br>0.4<br>0.36-0.44	149<br>0.23<br>0.2-0.27	11<br>0.017<br>0.009-0.031	160<br>0.25<br>0.22-0.29	1<br>0.002<br>0.00004-0.0087	637	
Males, 12-17 years	8<br>0.035<br>0.015-0.069	64<br>0.28<br>0.23-0.35	72<br>0.32<br>0.26-0.39	96<br>0.42<br>0.36-0.49	52<br>0.23<br>0.18-0.29	5<br>0.022<br>0.007-0.05	57<br>0.25<br>0.2-0.31	1<br>0.004<br>0.0001-0.024	226	0.4
Females, 12-17 years	24<br>0.058<br>0.038-0.086	127<br>0.31<br>0.26-0.36	151<br>0.3674<br>0.3111-0.4309	157<br>0.38<br>0.33-0.43	97<br>0.24<br>0.2-0.28	6<br>0.015<br>0.005-0.032	103<br>0.25<br>0.21-0.3	0	411	
Adults, ≥18 years	288<br>0.029<br>0.026-0.033	2288<br>0.23<br>0.22-0.24	2576<br>0.26<br>0.25-0.27	3708<br>0.37<br>0.36-0.38	3121<br>0.31<br>0.305-0.323	513<br>0.052<br>0.047-0.056	3634<br>0.37<br>0.36-0.38	14<br>0.0014<br>0.0008-0.0024	9932	
Adults, males, ≥18 years	112<br>0.053<br>0.044-0.063	505<br>0.24<br>0.22-0.26	617<br>0.29<br>0.27-0.31	738<br>0.35<br>0.33-0.37	629<br>0.3<br>0.28-0.32	128<br>0.06<br>0.051-0.072	757<br>0.36<br>0.34-0.38	2<br>0.0009<br>0.0001-0.0034	2114	**<0.001**
Adults, females, ≥18 years	176<br>0.023<br>0.019-0.026	1783<br>0.23<br>0.22-0.24	1959<br>0.25<br>0.24-0.26	2970<br>0.38<br>0.37-0.39	2492<br>0.32<br>0.31-0.33	385<br>0.05<br>0.045-0.054	2877<br>0.37<br>0.36-0.38	12<br>0.0015<br>0.0008-0.0027	7818	
18-24 years	40<br>0.042<br>0.03-0.057	313<br>0.33<br>0.3-0.36	353<br>0.37<br>0.34-0.4	350<br>0.37<br>0.34-0.4	213<br>0.23<br>0.2-0.25	27<br>0.029<br>0.019-0.041	240<br>0.25<br>0.22-0.28	0	943	
18-24 years, males	11<br>0.07<br>0.036-0.12	54<br>0.34<br>0.27-0.42	65<br>0.41<br>0.34-0.5	57<br>0.36<br>0.29-0.44	31<br>0.2<br>0.14-0.27	4<br>0.026<br>0.007-0.06	35<br>0.22<br>0.16-0.3	0	157	0.4
18-24 years, females	29<br>0.037<br>0.025-0.053	259<br>0.33<br>0.3-0.37	288<br>0.37<br>0.33-0.4	293<br>0.37<br>0.34-0.41	182<br>0.23<br>0.2-0.26	23<br>0.029<br>0.0186-0.0436	205<br>0.26<br>0.23-0.3	0	786	

Group	Vitamin D deficiency			Vitamin D insufficiency (suboptimal status)	Vitamin D sufficiency			Toxicity	Total, n	p
	Severe deficiency <25 nmol/L (<10 ng/mL)<br>n, PR*, 95% CI	Moderate deficiency 25-50 nmol/L (10-20 ng/mL)<br>n, PR*, 95% CI	Deficiency <50 nmol/L (<20 ng/mL) (total)<br>n, PR*, 95% CI	50–75 nmol/L (20-30 ng/mL)<br>n, PR*, 95% CI	Optimal 75–125 nmol/L (30-50 ng/mL)<br>n, PR*, 95% CI	Increased levels 125-250 nmol/L (50-100 ng/mL)<br>n, PR*, 95% CI	75-250 nmol/L 30-100 ng/mL (total)<br>n, PR*, 95% CI	>250 nmol/L (>100 ng/mL)<br>n, PR*, 95% CI		
25-44 years	124<br>0.023<br>0.019-0.027	1194<br>0.22<br>0.21-0.23	1318<br>0.24<br>0.23-0.25	2093<br>0.38<br>0.37-0.4	1809<br>0.33<br>0.32-0.34	264<br>0.048<br>0.043-0.054	2073<br>0.38<br>0.37-0.39	7<br>0.0013<br>0.0005-0.0026	5461	
25-44 years, males	47<br>0.048<br>0.036-0.064	247<br>0.25<br>0.23-0.28	294<br>0.3<br>0.27-0.33	345<br>0.35<br>0.32-0.38	291<br>0.3<br>0.27-0.33	45<br>0.046<br>0.034-0.061	336<br>0.34<br>0.31-0.38	0	975	**<0.001**
25-44 years, females	77<br>0.017<br>0.014-0.021	947<br>0.21<br>0.2-0.22	1024<br>0.23<br>0.22-0.24	1718<br>0.38<br>0.37-0.4	1518<br>0.34<br>0.32-0.35	219<br>0.049<br>0.043-0.056	1737<br>0.39<br>0.37-0.4	7<br>0.0016<br>0.0006-0.0032	4486	
45-64 years	91<br>0.033<br>0.027-0.04	589<br>0.21<br>0.2-0.23	680<br>0.25<br>0.23-0.26	1030<br>0.37<br>0.36-0.39	863<br>0.31<br>0.3-0.33	179<br>0.065<br>0.056-0.075	1042<br>0.38<br>0.36-0.4	5<br>0.0018<br>0.0006-0.0042	2757	
45-64 years, males	45<br>0.059<br>0.043-0.078	162<br>0.21<br>0.18-0.24	207<br>0.27<br>0.24-0.3	266<br>0.35<br>0.31-0.38	232<br>0.3<br>0.27-0.34	58<br>0.076<br>0.058-0.097	290<br>0.38<br>0.34-0.41	2<br>0.0026<br>0.0003-0.0094	765	**<0.001**
45-64 years, females	46<br>0.023<br>0.017-0.03	427<br>0.21<br>0.2-0.23	473<br>0.24<br>0.22-0.26	764<br>0.38<br>0.36-0.41	631<br>0.32<br>0.3-0.34	121<br>0.061<br>0.051-0.072	752<br>0.38<br>0.36-0.4	3<br>0.0015<br>0.0003-0.0044	1992	
65-79 years	28<br>0.04<br>0.027-0.057	175<br>0.25<br>0.22-0.28	203<br>0.29<br>0.26-0.32	239<br>0.34<br>0.31-0.38	221<br>0.32<br>0.28-0.35	37<br>0.053<br>0.037-0.072	258<br>0.37<br>0.33-0.41	2<br>0.0029<br>0.0003-0.0103	701	
65-79 years, males	7<br>0.034<br>0.015-0.075	38<br>0.2<br>0.15-0.27	45<br>0.24<br>0.18-0.31	60<br>0.32<br>0.25-0.39	67<br>0.36<br>0.29-0.43	16<br>0.085<br>0.049-0.135	83<br>0.44<br>0.37-0.52	0	188	0.06
65-79 years, females	21<br>0.041<br>0.026-0.062	137<br>0.28<br>0.23-0.3	158<br>0.31<br>0.27-0.35	179<br>0.35<br>0.3-0.39	154<br>0.3<br>0.26-0.34	20<br>0.039<br>0.024-0.06	174<br>0.34<br>0.3-0.38	2<br>0.0039<br>0.0005-0.014	513	
≥ 80 years	5<br>0.071<br>0.024-0.159	17<br>0.24<br>0.15-0.36	22<br>0.31<br>0.21-0.44	26<br>0.37<br>0.26-0.495	15<br>0.21<br>0.13-0.33	7<br>0.1<br>0.041-0.195	22<br>0.31<br>0.21-0.44	0	70	
≥ 80 years, males	2<br>0.069<br>0.009-0.23	4<br>0.14<br>0.039-0.32	6<br>0.21<br>0.08-0.4	10<br>0.34<br>0.18-0.54	8<br>0.28<br>0.13-0.47	5<br>0.17<br>0.059-0.36	13<br>0.45<br>0.26-0.64	0	29	0.2
≥ 80 years, females	3<br>0.073<br>0.015-0.199	13<br>0.32<br>0.18-0.48	16<br>0.39<br>0.24-0.56	16<br>0.39<br>0.24-0.56	7<br>0.17<br>0.72-0.32	2<br>0.049<br>0.006-0.165	9<br>0.22<br>0.1-0.38	0	41	

*PR – prevalence as proportions
n - number of participants
CI - the confidence interval
